# How effective are physiotherapy interventions in treating people with sciatica? A systematic review and meta-analysis

**DOI:** 10.1007/s00586-022-07356-y

**Published:** 2022-12-29

**Authors:** Lucy Dove, Gillian Jones, Lee Anne Kelsey, Melinda C. Cairns, Annina B. Schmid

**Affiliations:** 1 Nuffield Department of Clinical Neurosciences, John Radcliffe Hospital, The University of Oxford, West Wing Level 6, Oxford, OX3 9DU UK; 2 Oxford Spine Service, Nuffield Orthopaedic Centre, Oxford University Hospitals NHS Foundation Trust, Oxford, UK; 3 Department of Sport, Health Sciences and Social Work, Oxford Brookes University, Oxford, UK; 4 Oxford Health NHS Foundation Trust, Oxford, UK; 5 School of Health and Social Work, University of Hertfordshire, Hatfield, UK; 6 Physiocare Body Management, 6 Church St, Twyford, Reading, RG10 9DR UK

**Keywords:** Sciatica, Radicular pain, Lumbar radiculopathy, Physiotherapy, Systematic review

## Abstract

**Purpose:**

Physiotherapy interventions are prescribed as first-line treatment for people with sciatica; however, their effectiveness remains controversial. The purpose of this systematic review was to establish the short-, medium- and long-term effectiveness of physiotherapy interventions compared to control interventions for people with clinically diagnosed sciatica.

**Methods:**

This systematic review was registered on PROSPERO CRD42018103900. Cochrane Central Register of Controlled Trials (CENTRAL), CINAHL (EBSCO), Embase, PEDro, PubMed, Scopus and grey literature were searched from inception to January 2021 without language restrictions. Inclusion criteria were randomised controlled trials evaluating physiotherapy interventions compared to a control intervention in people with clinical or imaging diagnosis of sciatica. Primary outcome measures were pain and disability. Study selection and data extraction were performed by two independent reviewers with consensus reached by discussion or third-party arbitration if required. Risk of bias was assessed independently by two reviewers using the Cochrane Risk of Bias tool with third-party consensus if required. Meta-analyses and sensitivity analyses were performed with random effects models using Revman v5.4. Subgroup analyses were undertaken to examine the effectiveness of physiotherapy interventions compared to minimal (e.g. advice only) or substantial control interventions (e.g. surgery).

**Results:**

Three thousand nine hundred and fifty eight records were identified, of which 18 trials were included, with a total number of 2699 participants. All trials had a high or unclear risk of bias. Meta-analysis of trials for the outcome of pain showed no difference in the short (SMD − 0.34 [95%CI − 1.05, 0.37] *p* = 0.34, *I*^2^ = 98%), medium (SMD 0.15 [95%CI − 0.09, 0.38], *p* = 0.22, *I*^2 ^= 80%) or long term (SMD 0.09 [95%CI − 0.18, 0.36], *p* = 0.51, *I*^2 ^= 82%). For disability there was no difference in the short (SMD − 0.00 [95%CI − 0.36, 0.35], *p* = 0.98, *I*^2^ = 92%, medium (SMD 0.25 [95%CI − 0.04, 0.55] *p* = 0.09, *I*^2^ = 87%), or long term (SMD 0.26 [95%CI − 0.16, 0.68] *p* = 0.22, *I*^2^ = 92%) between physiotherapy and control interventions. Subgroup analysis of studies comparing physiotherapy with minimal intervention favoured physiotherapy for pain at the long-term time points. Large confidence intervals and high heterogeneity indicate substantial uncertainly surrounding these estimates. Many trials evaluating physiotherapy intervention compared to substantial intervention did not use contemporary physiotherapy interventions.

**Conclusion:**

Based on currently available, mostly high risk of bias and highly heterogeneous data, there is inadequate evidence to make clinical recommendations on the effectiveness of physiotherapy interventions for people with clinically diagnosed sciatica. Future studies should aim to reduce clinical heterogeneity and to use contemporary physiotherapy interventions.

**Supplementary Information:**

The online version contains supplementary material available at 10.1007/s00586-022-07356-y.

## Introduction

‘Sciatica’ is a broad term describing spinally referred pain of neural origin that radiates into the leg. The reported prevalence of sciatica varies widely (1.2–43%) [1], probably due to different diagnostic criteria, reflecting a heterogeneous patient population. Sciatica is a significant burden to healthcare and the economy, as a neuropathic component in low back pain it is not only linked to poorer quality of life, but also increases the already high costs of back pain by a further 67% [2]. Although prognosis is good for most patients, up to 45% continue to have symptoms for 12 months or longer [3].

Physiotherapy interventions such as exercise, manual therapy and psychological therapy are recommended in clinical guidelines for people with sciatica [4]. However, the available systematic reviews examining the effectiveness of physiotherapy interventions are at least ten years old. For example, study selection in the most recent systematic review comparing surgery versus conservative care ended in 2009 [5]. Their results could not be meta-analysed due to poor reporting and clinical heterogeneity. Similarly, a network-meta-analysis concluded its search in 2009 [6], finding no support for the effectiveness of exercise or traction while manipulation may be beneficial. However, the latter was based on a single study only. Prior to this, reviews specifically focusing on conservative management of sciatica were published in 2010 [7] and 2007 [8] and were unable to make strong conclusions on the superiority of any treatment. More recent reviews published in 2015 and 2016 were limited to a subset of physiotherapy interventions (e.g. physical activity versus surgery [9] and exercise versus advice to stay active [10]). A recent review [11] looked at a range of physiotherapy interventions, however the review did not include a meta-analysis.

Of note, sciatica is a heterogeneous condition with no agreed diagnostic criteria [12]. Most reviews to date make no reference to the clinical diagnosis of included study participants rendering it unclear whether patients had confirmed nerve involvement. The objective of this systematic review was therefore to assess the up-to-date evidence on the effectiveness of physiotherapy interventions compared with control interventions in people with clinically diagnosed sciatica.

## Methods

### Registration

The protocol was prospectively registered on PROSPERO (CRD42018103900). We are reporting our findings according to the updated guidance for the PRISMA guidance [13].

### Search strategy

We searched the following databases from inception to 29th January 2021: Cochrane Central Register of Controlled Trials (CENTRAL), CINAHL (EBSCO), Embase, PEDro, PubMed and Scopus. We also searched grey literature including trial registries (OpenGrey and clinicaltrials.gov). The search strategy was developed in consultation with a medical librarian and included keywords relating to sciatica, physiotherapy and randomised controlled trials (Supplemental Table 1).

### Study eligibility

Included studies were randomised controlled trials evaluating physiotherapy interventions compared to a control intervention in people with ‘sciatica’. Trials were eligible if study participants were diagnosed with spinally referred leg pain of neural origin. This diagnosis required at least one of the following: positive sensory, myotomal or reflex tests on neurological examination; positive neurodynamic test (e.g. straight leg raise, slump); imaging confirming spinal nerve compromise correlating with symptoms; presence of neuropathic pain determined with neuropathic pain questionnaires; electrodiagnostic testing or quantitative sensory testing suggesting nerve root involvement. Studies which either did not specify how the sciatica diagnosis was made or were simply using pain referral into the leg without other clinical tests confirming a neural component were excluded. No restrictions were made on sciatica symptom duration or intensity. Eligible trials must evaluate physiotherapy interventions such as exercise, manual therapy, physiotherapy-led education, or a combination of these. The control intervention needed to be a non-physiotherapy intervention (e.g. surgery, GP care, other non-physiotherapy care). The control intervention could also be placebo, sham or no intervention. No restrictions were made on language.

Trials that included participants with serious pathology (e.g. cancer, fracture, cauda equina), pregnant women or participants aged below 18 were excluded. Studies evaluating post-surgical physiotherapy were excluded. As recent reviews address the effectiveness of acupuncture for people with sciatica [14, 15], and acupuncture is not core physiotherapy practice in many countries, trials evaluating acupuncture were excluded.

### Study selection

Two reviewers (LD, GJ) screened studies independently. In a first step, titles and abstracts were screened, followed by full texts. Discrepancies were resolved by discussion and arbitration by a third reviewer (AS) if required.

### Quality assessment

Two reviewers (LD, LK) independently used the Cochrane Risk of Bias tool to assess study quality and risk of bias [16]. The tool was piloted on three excluded studies to test agreement of decision-making. Disagreements between reviewers were resolved by a third reviewer where required (GJ).

### Data extraction

Two reviewers (LD, LK) independently extracted data using a standardised form; consensus was used to resolve any discrepancies. The following information was extracted: author, year, country, characteristics of participants (e.g. age, duration, severity of symptoms), diagnostic criteria, physiotherapy and control intervention (type, frequency and duration). Outcomes were extracted at baseline and follow-up time points. Primary outcomes of interest were pain (e.g. numerical pain rating scale) and disability (e.g. Oswestry disability index). Secondary outcomes were global perceived effect, quality of life, change in neurological function, psychological parameters, adverse events, and dropout rates. Means, standard deviations and sample sizes were extracted for each outcome. If alternative summary statistics were provided, we transformed the data using recommended calculations [17]. If available, outcomes were extracted for different time points, and grouped according to time after randomisation as: short term (< 3 months); medium-term (> 3 months but < 12 months) or long-term (≥ 12 months). If multiple terms were reported within one period, the outcome closest to 7 weeks, 6 months and 12 months was used. When more than one body part was used to assess pain (e.g. leg and back pain), the highest score at baseline was used to reflect patients’ dominant symptoms. When more than one outcome measure was used within a trial for a specific outcome of interest, the outcome measure described by the trial authors as their primary measure was used.

### Data synthesis and analysis

If data were available for the same outcome measure from at least two trials, meta-analysis was performed using Revman v5.4. We calculated standardised mean differences (SMD) and 95% confidence intervals (CI). Random effects models with inverse variance weighting were used to account for the variability of included studies. Heterogeneity was calculated with *I*^2^ statistics and interpreted as follows: ‘might not be important’ (0–40%), ‘moderate’ (30–60%), ‘substantial ‘(50–90%), and ‘considerable’ (75–100%) [16]. We performed separate overall meta-analyses comparing physiotherapy interventions with control interventions for our primary outcomes of pain and disability.

We planned to perform a subgroup analysis according to type of physiotherapy interventions. However, this was impossible as interventions were too heterogeneous to pool. We performed a post hoc subgroup analysis comparing the effect of physiotherapy interventions according to the type of control intervention (minimal vs. substantial). Minimal intervention included advice/education only, GP care, or sham treatment. Substantial intervention included surgery, disc and epidural injections. Due to high risk of bias, we performed a post hoc sensitivity analysis, removing those studies where at least two parameters of risk of bias were rated as high. Results that could not be included in the meta-analysis were narratively described.

## Results

### Search

**Fig. 1:**
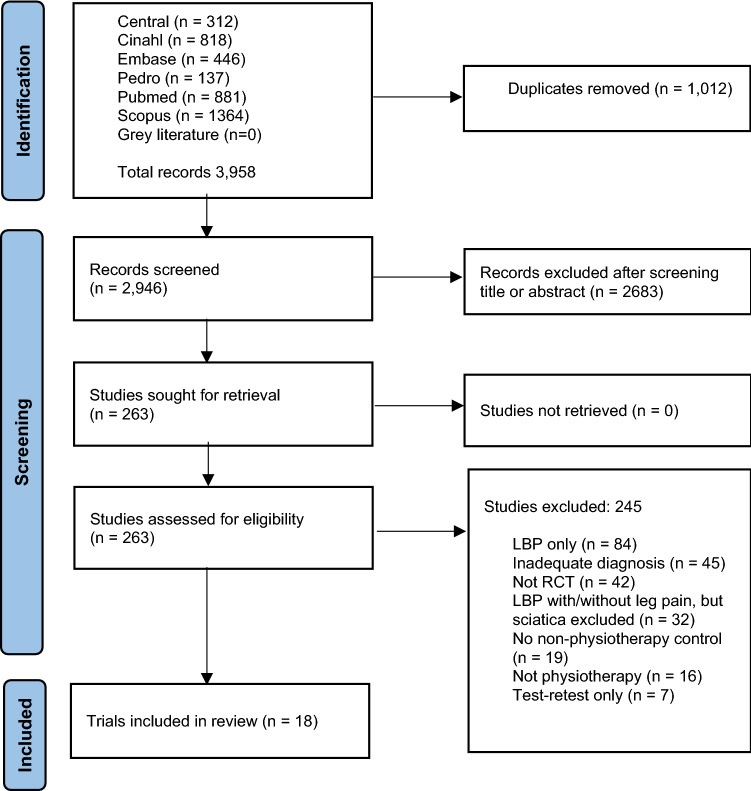
PRISMA flow diagram

The electronic database searches returned 3958 records. Duplicates and studies deemed ineligible from titles/abstracts were removed, leaving 263 full-text articles. Of those, 245 were discarded as they did not meet the inclusion criteria. A total of 18 studies were included in this systematic review (Fig. 1) [18–35].

### Risk of bias

**Fig. 2:**
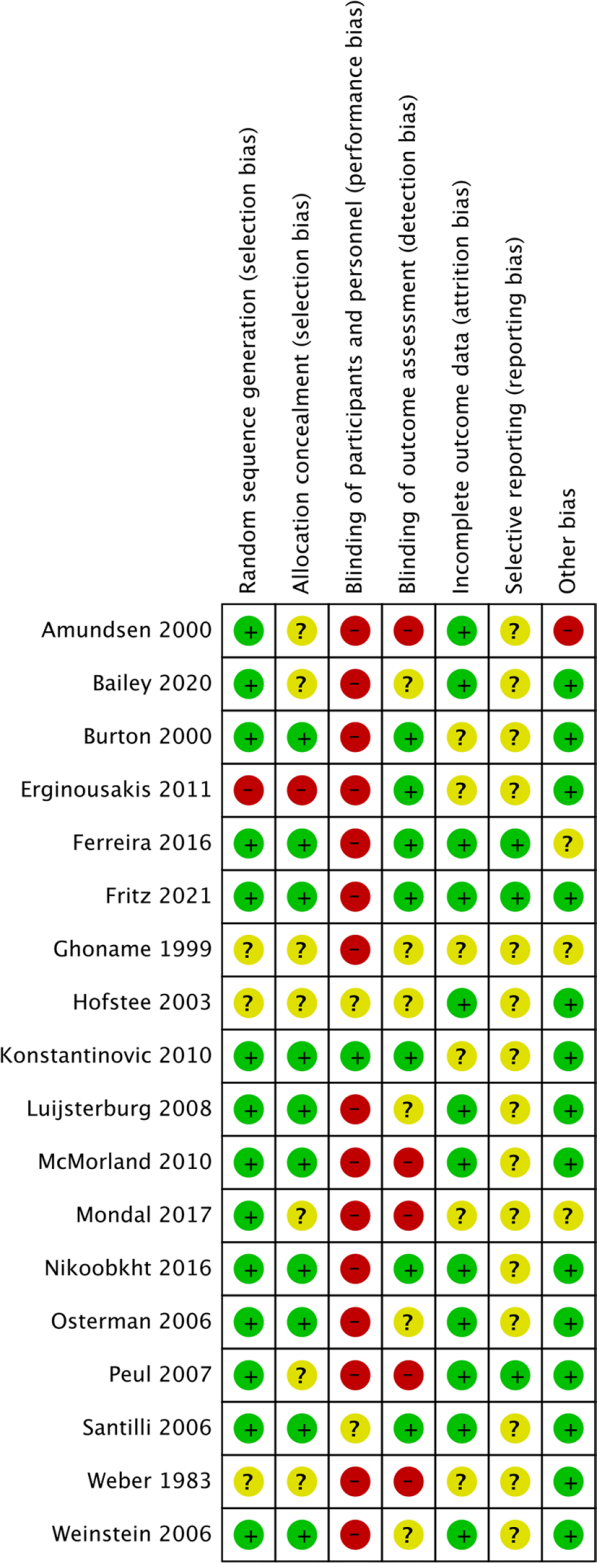
Risk of bias summary + low risk of bias? Unsure risk of bias—high risk of bias

Blinding of participants was understandably challenging to achieve in these trials, risk of performance bias was therefore high in 15 trials [18–20, 23, 25–35] and unclear in two trials [21, 24]. Detection bias was high or unclear in 11 [20, 21, 23, 25, 26, 29, 30, 32–35] of 18 studies (Fig. 2).

### Participants

Table 1 contains details of study characteristics. A total of 2699 participants were included, 1198 (44.4%) of them were female. According to data available from 13 trials [18–23, 26, 28, 30–33, 35] participants’ age ranged from a mean of 36.0 (SD 5.8) [28] to 48.38 (SD 6.39) years [30]. Baseline duration of sciatica was reported in eight trials, [18–22, 31–33], ranging from a mean of 1.8 (SD 1.3) weeks [21] to (median) 5.8 years (range 0.25–50) [18]. Pain severity at baseline was reported by 16 trials [18–24, 26–33, 35], ranging from a mean of 4.8 (SD 1.9) [19] to 8.0 (SD 1.8) [26] on an 11-point scale. The diagnostic criteria for sciatica used in the included studies are listed in Supplemental Table 2.

**Table 1:** Characteristics of included studies

Study Year Country	Number of participants Age in years (SD) Pain duration (SD) Pain severity (SD)	Physiotherapy intervention (group G1)	Control intervention (group G2)	Primary outcome measures and time points	Results G1*	Results G2*
*Physiotherapy vs. minimal intervention (7 RCTs)*						
Ferreira et al. [18] 2016 Brazil	*N* = 60 Age: G1 43.9(14.5) G2 40.3(12.9) Duration: G1 5.8yrs G2 2.0yrs Severity: NPRS (back) G1 5.5 (2.3) G2 5.1 (2.5) NPRS (leg) G1 6.1 (1.6) G2 6.1 (1.9)	Neurodynamic treatment. Passive or active movements. Education on nerve sensitisation. Grade III lumbar foramen opening mobilisations and neurodynamic sliders. Home exercise: one sliding and one tensioning technique.	Both groups advice to remain active, face-to-face. Advised to avoid prolonged rest, do not avoid daily-life activity, do not excessively brace muscles. Advised light activity and movement beneficial for pain.	NPRS (leg) 4w ODI 4w	Pain short 3.7(2.6) ODI short 20 (12)	Pain short 6.1(2.4) ODI short 23 (12)
Fritz et al. [19] 2021 USA	*N* = 220 Age: G1 40.0(11.2) G2 37.9(11.2) Duration: (days)G1 35.8(25.6) G2 35.9(26.8) Severity: NPRS (back) G1 5.1(1.8) G2 4.8(1.9) NPRS (leg) G1 4.3(2.2) G2 3.8(2.2)	Physical therapy within 3 days of assignment. Exercise and manual therapy in each session. Written directions and instructed to do assigned exercises at home.	Medication and imaging at discretion of primary care provider. Given copy of The Back Book, about favourable prognosis, and importance of remaining active.	NPRS (back) 4w, 6m, 12m ODI 4w, 6m, 12m	Pain short 2.4, (95%CI 2, 2.8) Pain med 2.6, (95%CI 2.2, 3) Pain long 2.3, (95%CI 1.9, 2.7) Disability short 19.9 (95%CI 17.2, 22.7) Disability med 14.5, (95%CI 11.6, 17.3) Disability long 14.4, (95%CI 11.5, 17.4)	Pain short 3.9 (95%CI 3.5, 4.3) Pain med 3.3, (95%CI 2.9, 3.7) Pain long 3.3, (95%CI 2.9, 3.7) Disability short 28.1 (95%CI 25.4, 30.8) Disability med 19.8, (95%CI 17.0, 22.7) Disability long 19.2, (95%CI 16.3, 22.0)
Ghoname et al. [20] 1999 USA	*N* = 64 Age: G1 43(19) G2 43(19) Duration: (months) 21(9) Severity: NPRS (leg) G1 7 (1.9) G2 6.6 (1.9)	Standard TENS therapy: 4 electrode pads in standardised pattern, stimulated at 4Hz, pulse duration 0.1s. Intensity adjusted to maximum tolerated without producing muscle contractions.	Sham-PENS: placement of 10 acupuncture-like needle probes in identical montage to PENS treatment. However, no electrical stimulation was applied to the probes.	NPRS (leg) 3w VAS physical activity 3w	Pain short 5.4 (1.9) Disability short 4.5 (1.7)	Pain short 6.1 (1.9) Disability 5.5 (2.1)
Hofstee et al. [21] 2003 The Netherlands	N = 250 Age: G1 38(9.5) G2 38(9.5) Duration: (wks)G1 1.8(1.3) G2 1.9(1.2) Severity: VAS G1 60.9 (20.1) G2 65.5 (18.5)	Physiotherapy (exercises, advice, hydrotherapy, home exercise programme).	Continuation of normal activities as much as possible (modify duration, intensity, and frequency according to pain).	Pain VAS 2m, 6m QDS 2m, 6m	Pain short 23.9 (IQR 20,60) Pain med 14.1 (IQR 29,70) Disability short 29.7 (IQR 8.5, 44) Disability med 21.4 (IQR 20,51)	Pain short 23.4 (IQR 17,64) Pain med 12.9 (IQR 26,66) Disability short 31.1 (IQR 10, 42) Disability med 22 (IQR 18,52)
Konstantinovic et al. [22] 2010 Serbia	*N* = 364 Age: G1 43.5(7.7) G2 41.87 (8.37) Duration: < 4w Severity: (leg) G1 78.5(3.14) G2 74.7(6.05)	Active low-level laser therapy behind involved spine segment using stationary skin-contact method. 5x weekly, total of 15 treatments, frequency 5000Hz, dose 3J/cm^2^; treatment time 150 seconds.	Placebo laser treatment applied in same manner as active device by identical device that was deactivated by member of Institute for Physics.	VAS leg 3w ODI 3w	Pain short median 34 (IQR 30.5; 38) Disability short median 20 (IQR 19;21)	Pain short median 54 (IQR 50;56) Disability short median 22 (IQR 20;24)
Luijsterburg et al. [23] 2008 The Netherlands	*N* = 135 Age: G1 42 (10) G2 43 (12) Duration: (inclusion) < 6wks Severity: NRS G1 6.3(2.2) G2 6.3(2.2)	Exercise therapy, advice, guidance: return to activity despite pain, type/content of exercise left to PT. Passive treatment not allowed.	GP care according to clinical guideline, information, advice and, if necessary, pain medication prescribed.	NRS leg 6w, 12w, 12m RDQ 6w, 12w, 12m	Pain short 3.3 (2.67) Pain med 2.4 (2.96) Pain long 1.9 (2.82) Disability short 10.6 (6.67) Disability med 8.2(7.11) Disability long 5.9(6.37)	Pain short 3 (2.67) Pain med 2.6 (2.96) Pain long 2.6 (2.82) Disability short 8.8 (6.67) Disability med 6.9(7.11) Disability long 6.3(6.37)
Santilli et al. [24] 2006 Italy	*N* = 102 Age: (inclusion) 18 to 65 Duration: (inclusion) < 10d Severity: VAS G1 6.4(0.9) G2 6.4(0.8)	Active manipulations according to protocol by chiropractor including soft tissue manipulations and rotational thrust away from greatest restriction.	Simulated manipulations, soft muscle pressing not specific patterns, not rapid thrusts. Chiropractors as G1.	Local pain reduction 90d, 180d; Radiating pain reduction 90d, 180d; Local pain-free 90d, 180d; Radiating pain-free 90d, 180d.	Pain med (n) radiating pain reduction 48, % pain free 100 Pain long (n) radiating pain reduction 48, % pain free 100	Pain med (n) radiating pain reduction 39, % pain free 81 Pain long (n) radiating pain reduction 40, % pain free 83
*Physiotherapy vs. substantial intervention (11 RCTS)*						
Amundsen et al. [25] 2000 Norway	*N* = 31 Age: G1 83% 40-70; G2 84% 40-70 Duration not reported Severity: G1 28% mod, 72% severe G2 46% mod, 54% severe	1-month inpatient stay, 3-point hyperextension thoracolumbar brace. Physiotherapy when home, walking and stabilising exercises, kyphotic position encouraged.	Partial/total laminectomy, medial facetectomy/discectomy and/or removal of osteophytes. 1–2 days post-op brace, physiotherapy as previously.	Subjective report 6m, 12m	Pain med (n): No/light 2 (cross 5) Mod 5 (cross 4) Severe 1 (cross 1) Pain long (n): No/light 1 (cross 1); Mod 7 (cross 3); Severe 0 (cross 4)	Pain med (n) No/light 2, Moderate 11, Severe 0 Pain long (n): No/light 5, Moderate 7, Severe 0
Bailey et al. [26] 2020 Canada	*N* = 128 Age: G1 37.1(11.9) G2 38 (8.3) Duration: (inclusion) 4-12m Severity: VAS back G1 6.5(2.8) G2 6.7(2.6) VAS leg G1 8.0(1.8) G2 7.7(2.0)	Education regarding activity and exercise, use of oral analgesics. Active physiotherapy provided at the discretion of PT. Optional epidural, 2^nd^/3^rd^ injection at discretion of physician.	Microdiscectomy fellowship-trained spine surgeon open/minimal access approach, loupe/microscope assistance.	VAS leg 6m, 12m ODI 6m, 12m	Pain med 5.2 (0.4SE) Pain long 4.7 (0.4SE) Disability med 33.7(2.3SE) Disability long 34.7(2.4SE)	Pain med 2.8 (0.4SE) Pain long 2.6 (0.4SE) Disability med 22.8 (2.3SE) Disability long 22.9 (2.3SE)
Burton et al. [27] 2000 UK	*N* = 40 Age: 41.9 (10.6) no reports per group Duration not reported Severity: 7 pt scale G1 3.79(1.62) G2 4.05(1.28)	Soft tissue stretching of lumbar/buttock muscles, low-amplitude passive manoeuvres lumbar spine. Clinical discretion re: manipulation. Advice: continue normal activity, encouraged return work.	General anaesthetic, single injection of chymopapain into nucleus of disc and bupivacaine. Discharge following day to usual care of family doctor.	7-point scale back pain 6w, 12m RDQ 6w, 12m	Pain short 2.68 (1.6) Pain long 2.27 (1.53) Disability short 7.79 (6.65) Disability long 5.87 (5.96)	Pain short 3.58 (0.97) Pain long 2.87 (1.36) Disability short 11 (5.69) Disability long 7.27 (6.65)
Erginousakis et al. [28] 2011 Greece	*N* = 62 Age: G1 36(5.8) G2 38(4.2) Duration not reported Severity: NVS G1 6.9(1.9) G2 7.4 (1.4)	Conservative therapy including education, counselling, physical therapy, NSAIDs, muscle relaxants, analgesics.	Fluoroscopically guided percutaneous disc decompression.	NVS 3m, 12m	Pain short 0.9 (2) Pain long 4 (3.4)	Pain short 3.0 (2.4) Pain long 1.7 (2.4)
McMorland et al. [29] 2010 Canada	*N* = 40 Age: G1 42.4 G2 41.5 (SD unreported) Duration: (inclusion) > 3m Severity: McGill PRI(R) G1 28.7 (17.4) G2 32.5 (12.9)	Spinal manipulative therapy at discretion of treating clinician, ice or heat, information, education, intro to rehab exercises. Core stability exercise, emphasis on technique.	Surgical microdiscectomy, hospital for 1-2 days. Analgesia for 10 days and advised to avoid heavy lifting, bending or twisting for 6-8 weeks.	McGill PRI(R) 6w RMDQ 6w	Pain short 21.7 (13.7) Disability short 9.5 (6.0)	Pain short 18.4 (16.3) Disability short 9.4 (6.4)
Mondal et al. [30] 2017 India	*N* = 60 Age: G1 48.38 (6.39) G2 42.11 (8.58) Duration: > 3m Severity: (inclusion) > 5 NRS	Spine extension exercises.	Single transforaminal epidural steroid injection with methylprednisolone acetate (20mg and 0.25% bupivacaine (total 2ml) and spine extension exercises.	NRS 1m ODI 1m	Pain short 5.03 (2.06) Disability short 56.94 (23.8)	Pain short 3.11 (2.06) Disability short 34.79 (23.8)
Nikoobakht et al. [31] 2016 Iran	*N* = 177 Age: G1 38.0(9.0) G2 37.6(7.3) Duration: (m)G1 25.9(8.6) G2 18.6(12.0) Severity: VAS G1 7.4(1.5) G2 7.6(1.5)	Bed rest, active physical therapy, education & counselling, home exercises, spinal manipulation, analgesics, muscle relaxants, NSAIDs & local injections.	Percutaneous disc decompression under moderate sedation. Graduated return to normal activity in the 2 wks following procedure.	VAS 1m, 3m, 12m ODI 1m, 3m, 12m	Pain short 6.94 (2.27) Pain med 6.6 (2.67) Pain long 6.14 (3.07) Disability short 38.75 (13.27) Disability med 36.76 (15.39) Disability long 35.29 (16.43)	Pain short 5.83 (3.25) Pain med 5.36 (3.43) Pain long 4.68 (3.58) Disability short 28.50 (17.02) Disability med 19.87 (15.49) Disability long 10.84 (12.75)
Osterman et al. [32] 2006 Finland	*N* = 56 Age: G1 38(7); G2 37(7) Duration (d): G1 60(21); G2 77(32) Severity: VAS G1 57(21); G2 61(20)	Encouraged early physical activity within pain limits, instruction on isometric exercises.	Microdiscectomy within 2 wks of randomisation. Analgesia per individual requirements. Isometric exercise pre and post-op. Active physiotherapy	VAS leg 6w, 6m, 12m ODI 6w, 6m,12m	Pain short 25(27) Pain med 18 (29) Pain long 9 (19) Disability short 22 (16) Disability med 12 (15) Disability long 11(14)	Pain short 12(20) Pain med 9 (20) Pain long 6 (11) Disability short 16 (16) Disability med 8 (12) Disability long 10 (13)
Peul et al. [33] 2007 The Netherlands	*N* = 283 Age: G1 43.5(9.6) G2 41.7(9.9) Duration: (wks) G1 9.5(2.1) G2 9.4(2.4) Severity: VAS back G1 30.8(27.7) G2 33.8(29.6) VAS leg G1 64.4(21.2) G2 67.2(27.7)	GPs provided prolonged conservative treatment. Informed favourable prognosis, website informed natural course of illness & expectation of recovery. Patients fearful of movement referred to physiotherapy.	Surgery within 2 weeks to remove symptomatic disc herniation. Rehabilitation at home by physiotherapists standardised exercise protocol. Advice to resume activity.	VAS leg 8w, 6m, 12m RDQ 8w, 6m, 12m	Pain short 27.9 (1.9SE) Pain med 14.5 (1.9SE) Pain long 11 (1.9SE) Disability short 9.2 (0.5SE) Disability med 4.8 (0.5SE) Disability long 3.7 (0.5SE)	Pain short 10.2 (1.9SE) Pain med 8.4 (1.9SE) Pain long 11 (1.9SE) Disability short 6.1 (0.5SE) Disability med 4 (0.5SE) Disability long 3.3 (0.5SE)
Weber et al. [34] 1983 Norway	*N* = 126 Age: G1 41.7 G2 40 (SD not reported) Duration not reported Severity not reported	Wk 1 strict bed rest, moderate isometric exercises, analgesics. Wk 2 partial bed rest, gradual increase in exercise. Group ‘back school’ continued.	Surgical extradural removal of herniated mass of cartilage, out of bed day 1 post-op and discharge home 7-9d post-op without further treatment.	Patient subjective report of improvement as good/fair/poor/bad 12m.	Long term (n): Good 16 (8 cross); Fair 24 (4 cross; Poor 9 (4 cross); Bad 0 (1 cross)	Long term(n): Good 39 (0 cross); Fair 15 (1 cross); Poor 5 (0 cross); Bad 0
Weinstein et al. [35] 2006 USA	*N* = 501 Age: G1 43(11.3) G2 41.7(11.8) Duration: (inclusion) > 6wks Severity: SF-36 G1 26.7(17.4) G2 27.1(18.5)	Usual care, at least active physical therapy, education/counselling, home exercise, NSAIDs if tolerated. Individualised treatment tracked prospectively.	Standard open discectomy with examination of the involved nerve root. General/local anaesthetic. Nerve root decompressed.	SF-36 3m, 12m ODI 3m, 12m	Pain med 27.6 (1.8SE) Pain long 36.9 (1.8SE) Disability med 25 (1.6SE) Disability long 18.9 (1.6SE)	Pain med 30.5 (1.9SE) Pain long 39.7 (1.8SE) Disability med 21.5 (1.7SE) Disability long 16.9 (1.7SE)

*RCT* randomised controlled trial; *G* group; *SD* standard deviation; *SE* standard error; *NPRS* numeric pain rating scale; *ODI* Oswestry disability scale; *VAS* visual analogue scale; *QDS* Quebec disability scale; *GP* general practitioner; *RDQ* Roland disability scale; *NVS* numeric visual scale; *APS* Aberdeen pain scale; *McGill PRI(R)* McGill pain rating index rank value; *TENS* transcutaneous electrical nerve stimulation; *PENS* percutaneous electrical nerve stimulation; *PT* physiotherapist/physical therapist; *IQR* interquartile range; *CI confidence interval*; *med* medium; cross crossover; *m* month; *wk* week; *d* days

*Data are reported as mean (SD) unless stated otherwise

### Physiotherapy intervention

Physiotherapy interventions varied considerably in the components included which prevented the preplanned subgroup analyses according to type of physiotherapy. Eleven trials included exercise [18, 19, 21, 23, 25, 29–32, 34, 35]. Type of exercise was most often unspecified or was at the discretion of the treating physiotherapist. Four studies made specific reference to neurodynamic exercise, [18] core stability [29], extension exercises [30] and isometric exercise [32]. Eleven trials provided advice or education as part of the physiotherapy intervention [18, 21–23, 26–29, 32, 33, 35] with the most common advice to continue normal activity. Five studies used manual therapy or manipulations [19, 24, 27, 29, 31]. The frequency and duration of physiotherapy interventions were unreported in seven trials [23, 25, 29, 30, 33–35]. Where duration was reported, it ranged from 2 weeks [18] to 6 months [26]. Further details on physiotherapy interventions are available in Tables 1 and 2.

**Table 2:** Components of physiotherapy interventions

Study	Exercise	Advice/Education	Manual therapy	Home exercise	Oral analgesia/ neuropathic	Frequency/duration of physiotherapy intervention	Additional interventions/ adjuncts
*Physiotherapy vs. minimal intervention (7 RCTs)*							
Ferreira et al. [18]	√	√		√		4 treatment sessions over 2 weeks	
Fritz et al. [19]	√		√	√		6-8x during 4 wks, 2x each wk during first 2 wks and 1-2x in wks 3&4. Home exercises every 4-5 hours days between sessions.	
Ghoname et al. [20]						30 mins 3x weekly for 3 weeks	TENS therapy, 4 x 2.5cm cutaneous pads at 4Hz, pulse duration 0.1s
Hofstee et al. [21]	√	√		√		Twice weekly, minimum 4 wks maximum 8 wks	Hydrotherapy
Konstantinovic et al. [22]		√			√	5x weekly for a total of 15 treatments	Low level laser therapy, 5000 frequency, 100mW, 3J
Luijsterburg et al. [23]	√	√				Not reported	
Santilli et al. [24]			√			5 days per week for 30 days	
*Physiotherapy vs. surgical (11 RCTS)*							
Amundsen et al. [25]	√					Not reported	3m inpatient stay, 3-point thoracolumbar hyperextension brace
Bailey et al. [26]		√			√	Spinal specialist medications, education & assessment of response to treatment on 6-wk basis min of 6m	Active physiotherapy at discretion of physiotherapists (number unspecified). Optional epidural injection
Burton et al. [27]		√	√			12 weeks maximum	Soft tissue stretching of lumbar and buttock muscles
Erginousakis et al. [28]		√			√	Mean duration 22 days (range 7–35 days)	
McMorland et al. [29]	√	√	√			Not reported	Ice or heat
Mondal et al. [30]	√				√	Not reported	
Nikoobakht et al. [31]	√		√	√	√	20 sessions, 12 weeks	Bed rest, local injections
Osterman et al. [32]	√	√				3 times (at follow-ups 6wk, 3m, 12m)	
Peul et al. [33]		√				Not reported	Patients fearful of movement referred to physiotherapy (number unspecified)
Weber et al. [34]	√					Not reported	Strict bed rest week 1, partial bed rest week 2. Group lessons in ‘back school’
Weinstein et al. [35]	√	√		√	√	Not reported	

*m* month; *wk* week

### Control intervention

Minimal intervention included advice to stay active [18] provision of a Back Book education booklet [19], bedrest or advice to continue normal activity [21], sham electrical nerve stimulation [20], sham laser therapy [22], GP care [23] or simulated manipulations [24]. Substantial interventions involved surgery such as microdiscectomy or discectomy [26, 29, 32–35], or decompression [25, 28, 31]. One study compared epidural injection with extension exercises [30] and one compared chemonucleolysis disc injection [27] with physiotherapy.

### Reporting of outcomes

Fifteen studies reported pain as a continuous outcome [18–23, 26–33, 35]. The three remaining studies reported a categorical outcome [24, 25, 34]. Fourteen studies reported a measure of disability [18–23, 26, 27, 29–33, 35]. Secondary outcome measures were not always reported (Supplemental Table 3). One trial reported treatment adherence [18]. Adverse events were unreported in seven trials [20, 23–25, 28, 30, 34]. Of these, five [20, 23–25, 34] pre-date publication of Consort Guidelines [36] which includes reporting of adverse events. Supplemental Table 4 summarises details of the adverse events, which were less frequent with physiotherapy interventions than substantial control interventions. Dropout rates were unreported in three trials [20, 28, 29].

### Overall meta-analysis on physiotherapy versus control intervention

**Fig. 3:**
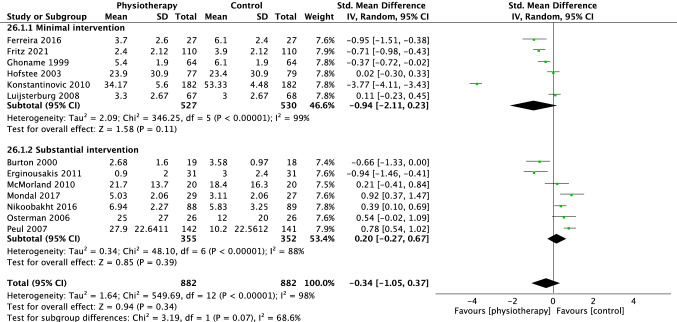
Forest plot pain short term (< 3 months)

**Fig. 4:**
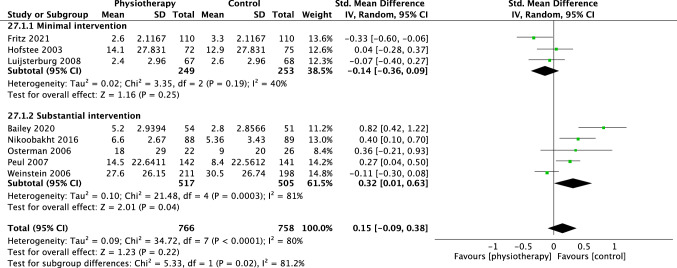
Forest plot pain medium term (> 3 months < 6 months)

**Fig. 5:**
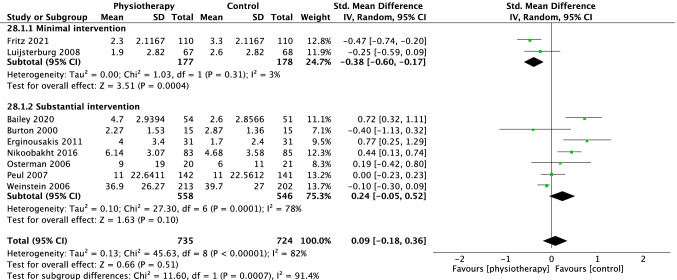
Forest plot pain long term (> or = 12 months)

For pain, 13 trials were included in the overall meta-analysis comparing physiotherapy versus all control interventions at short term, eight trials at medium term and nine trials at long-term time points. There was no difference in effectiveness of physiotherapy versus control interventions at short term (SMD − 0.34 [95%CI − 1.05, 0.37] *p* = 0.34, *I*^2^ = 98%, Fig. 3), medium term (SMD 0.15 [95%CI − 0.09, 0.38], *p* = 0.22, *I*^2^ = 80%, Fig. 4) and long term (SMD 0.09 [95%CI − 0.18, 0.36], *p* = 0.51, *I*^2^ = 82% Fig. 5).

**Fig. 6:**
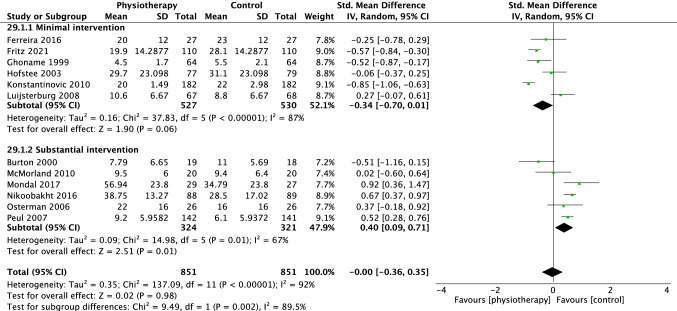
Forest plot disability short term (< 3 months)

**Fig. 7:**
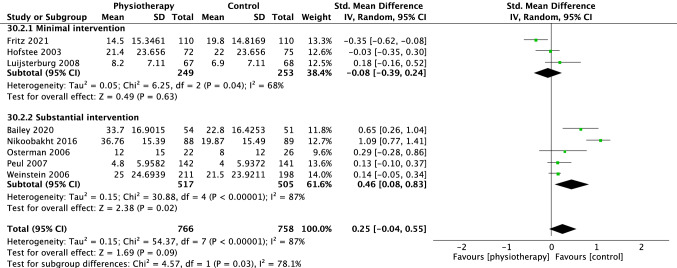
Forest plot disability medium term (> 3 months < 6 months)

**Fig. 8:**
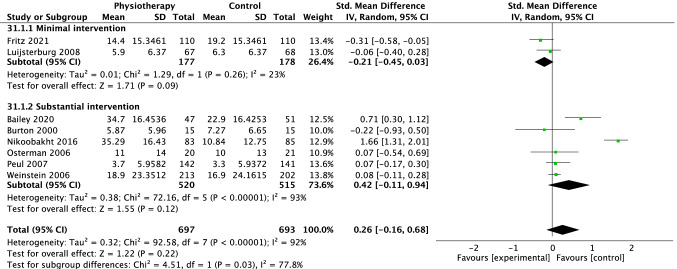
Forest plot disability long term (> or = 12 months)

For disability, 12 trials were included in the overall meta-analysis at short term, eight trials at medium term and eight trials at long term. There was no difference in effectiveness of physiotherapy versus control interventions at short (SMD − 0.00 [95%CI − 0.36, 0.35], *p* = 0.98, *I*^2^ = 92%, Fig. 6), medium (SMD 0.25 [95%CI − 0.04, 0.55] *p* = 0.09, *I*^2^ = 87%, Fig. 7) and long term (SMD 0.26 [95%CI − 0.16, 0.68] *p* = 0.22, *I*^2^ = 92%, Fig. 8).

### Subgroup analysis on physiotherapy versus minimal intervention

For pain, six studies comparing physiotherapy with a minimal intervention were included in the subgroup analysis at short term, [18–23] three at medium [19, 21, 23] and two at long term [19, 23]. There were no group differences at short (SMD − 0.94 [95%CI − 2.11, 0.23] *p* = 0.11 *I*^2^ = 99%, Fig. 3) or medium-term (SMD − 0.14 [95% CI − 0.36, 0.09] *p* = 0.25, *I*^2^ = 40%, Fig. 4). However, there was a small effect (SMD − 0.38 [95% CI − 0.60, − 0.17, *p* = 0.0004, *I*^2^ = 3%], Fig. 5) in favour of physiotherapy interventions for pain reduction at the long-term time point.

One study [24] could not be meta-analysed due to insufficient data. Nonetheless, the results were broadly consistent with the meta-analysis. Santilli et al. [24] reported number of participants with reduction in radiating pain. At medium term, 48 participants (100%) of the physiotherapy group (spinal manipulation) reported reduction in radiating pain compared with 39 (81%) of those in the sham group. At long-term follow-up, 48 patients (100%) of the physiotherapy group continued to report reductions in radiating pain compared with 40 participants (83%) in the sham group.

For disability, six trials were meta-analysed comparing physiotherapy with minimal intervention at short term, [18–23] three at medium [19, 21, 23] and two trials at long term [19, 23]. No group differences were observed at short (SMD − 0.34 [95%CI − 0.70, − 0.01] *p* = 0.06, *I*^2^ = 87%, Fig. 6) medium, (SMD − 0.08 [95% CI − 0.39, 0.24] *p* = 0.63, *I*^2^ = 68%, Fig. 7) or long-term time points (SMD − 0.21 [95% CI − 0.45, 0.03] *p* = 0.09, *I*^2^ = 23%, Fig. 8). The Santilli [24] study did not report a measure of disability at any time point. Overall, these findings suggest that physiotherapy interventions are slightly more effective than minimal treatment for pain in the long term but not at short or medium term.

### Subgroup analysis on physiotherapy versus substantial intervention

Eleven trials compared physiotherapy with substantial control intervention. Nine [26–33, 35] were included in the subgroup analysis for pain. There was no difference between physiotherapy and substantial intervention for the outcome of pain in the short (SMD 0.20 [95%CI − 0.27, 0.67] *p* = 0.39, *I*^2^ = 88%, Fig. 3) or long term (SMD 0.24 [95%CI − 0.05, 0.52], *p* = 0.10, *I*^2^ = 78%, Fig. 5). There was a small effect in favour of substantial intervention in the medium term (SMD 0.32 [95%CI 0.01, 0.63], *p* = 0.04, *I*^2^ = 81%, Fig. 4).

Two trials reported results that were not possible to incorporate in either meta-analysis [25, 34]. Amundsen [25] reported improvements in both the physiotherapy and surgical arms, however groups were not statistically compared. Weber [34] reported slightly higher rates of improvement in surgical compared to physiotherapy interventions at one year.

Seven trials were included in the meta-analysis for the outcome of disability [26, 27, 30–33, 35]. There was a small effect in favour of substantial interventions at short (SMD 0.40 [95%CI 0.09, 0.71] *p* = 0.01, *I*^2^ = 67%, Fig. 6) and medium term (SMD 0.46 [95%CI 0.08, 0.83], *p* = 0.02, *I*^2^ = 87%, Fig. 7) but no difference in the long term (SMD 0.42 [95%CI − 0.11, 0.94], *p* = 0.12, *I*^2^ = 93%, Fig. 8).

#### Sensitivity analysis

Four studies with high risk of bias in at least 2 parameters [28–30, 33] were removed from the meta-analysis. The sensitivity analyses revealed consistent results for all comparisons apart from the subgroup comparison of physiotherapy versus substantial control intervention (Supplemental Figs. 1–6). With the removal of high risk of bias studies, the effect on pain at medium term and on disability at short term favouring substantial interventions was no longer present (Supplemental Figs. 2 and 4).

## Discussion

This systematic review, including 18 studies and 2699 participants with a clinical diagnosis of sciatica suggests that physiotherapy interventions are only better than minimal interventions in reducing pain at long-term time points. Physiotherapy interventions are less effective than substantial interventions (e.g. surgery) in reducing pain at medium term and disability at short- and medium-term time points. However, heterogeneity was considerable in most meta-analyses, and confidence intervals were large, indicating substantial uncertainly surrounding the precision of these estimates. The favourable results for substantial intervention for pain in medium term and disability in short term did not persist following sensitivity analyses removing studies with high risk of bias. The currently available literature therefore provides insufficient evidence to support strong recommendations for physiotherapy interventions in the treatment of people with sciatica.

This systematic review reflects a wider collective inability to show significant benefit of non-surgical treatments for people with sciatica. Pharmacological options fail to demonstrate effects beyond placebo [37], including non-steroidal anti-inflammatories [38], anti-convulsants [39], anti-depressants [40] or opioids [4, 41]. Epidural cortisone injections have small effect sizes and short-term benefits [42]. These findings are disappointing given the clear need for effective conservative interventions voiced by patients [43].

Apart from the possibility that physiotherapy is indeed not effective for patients with sciatica, there are multiple possible reasons for the lack of evidence. The physiotherapy interventions used in the 11 trials comparing physiotherapy with substantial interventions are not all considered contemporary in line with current clinical guidelines [4]. This is a reflection of a lack of recent physiotherapy trials, with only four of the 11 studies published in the last decade [26, 28, 30, 31]. Current clinical guidelines recommend group exercise and continuation of normal activities; however, bedrest was a component of the conservative treatment arm in two trials [28, 34]. The UK NICE Guidelines [4] find no evidence supporting the use of corsets or belts, but these were a core component in another trial [25] conducted before publication of these guidelines. The physiotherapy interventions are highly heterogeneous and remain unclear in several studies. The Bailey study [26] leaves physiotherapy interventions at the discretion of the treating clinician, and the Peul study [33] refers people to physiotherapy only if they are fearful of movement, leaving uncertainty about how many participants in those trials had active physiotherapy treatment. It could also be argued that patients deemed suitable for surgery are likely to represent a specific subgroup that may be less amenable to physiotherapeutic interventions (e.g. with intractable pain or neurological deficit). Indeed, two trials comparing physiotherapy interventions with surgery included patients who had already failed conservative treatment [28, 29], raising serious concerns that physiotherapy interventions could possibly succeed in such a population.

A further challenge to progress in treatment is the diagnosis of sciatica itself [44]. There is no agreed definition for sciatica, reflected in the wide range of definitions used in clinical trials [12], including our review. The broad term ‘sciatica’ comprises radiculopathy, radicular pain, or somatic referred pain. The differing patient populations bring clinical heterogeneity to most meta-analyses. Unfortunately, the high heterogeneity among studies reduces the confidence in our results. Together with previous systematic reviews with inconclusive findings, our results question the value of continuing to perform clinical trials in heterogeneous groups of patients. Although subgrouping according to risk stratification showed promise in the management of people with non-specific low back pain [45], this has failed in patients with sciatica [46]. Subgrouping using a mechanism-based approach shows promising signals in patients with neuropathic pain of different aetiologies [47], but has yet to be examined in sciatica.

The risk of bias analysis highlights areas of improvement for future trials. Performance bias is the area with the highest risk of bias. Although recent studies have shown that blinding of participants is possible [48], it is not easy to eradicate this bias where the intervention is a physical one such as surgery or physiotherapy. The main area that could easily be addressed is detection bias. Blinding outcome assessment would have reduced overall risk of bias in four studies.

### Strengths and limitations

The main strength of this review was the strict inclusion criteria based on clinical diagnosis confirming spinally referred leg pain of neural origin. A consequence of the tight inclusion criteria is the exclusion of 45 studies due to inadequate information on diagnosis of sciatica. As a result, our data reflect outcomes in patients with true nerve involvement. Insufficient reporting and low number of studies prevented a subgroup analysis according to type of physiotherapy intervention. Future trials with physiotherapy intervention should adhere to the TIDieR framework to fully describe the complexity of the intervention [49].

## Conclusion

In summary, in patients with clinically diagnosed sciatica, physiotherapy interventions trialed to date provide inadequate evidence to make specific recommendations on their effectiveness in reducing pain or disability. The lack of convincing evidence may be due to several factors including incomplete trial reporting, clinical, methodological, and statistical heterogeneity, and trials lacking high methodological quality. Rather than continuing to perform trials in the heterogeneous population of ‘sciatica’, future studies should focus on reducing clinical heterogeneity, using contemporary physiotherapy interventions and high methodological quality to hopefully end the roadblock of discovery on the most effective physiotherapy interventions for these patient populations.

## Supplementary Information

Below is the link to the electronic supplementary material.

Supplementary file1 (DOCX 5446 kb)

Supplementary file2 (DOCX 45 kb)
