# Supplementary material for: How effective are physiotherapy interventions in treating people with sciatica? A systematic review and meta-analysis
Source: Eur Spine J. 2022 Dec 29;32(2):517–33. doi: 10.1007/s00586-022-07356-y (PMC9925551; doi:10.1007/s00586-022-07356-y)
Supplement: Supplementary file 1 — Supplementary file1 (DOCX 5446 kb) [file 586_2022_7356_MOESM1_ESM.docx]

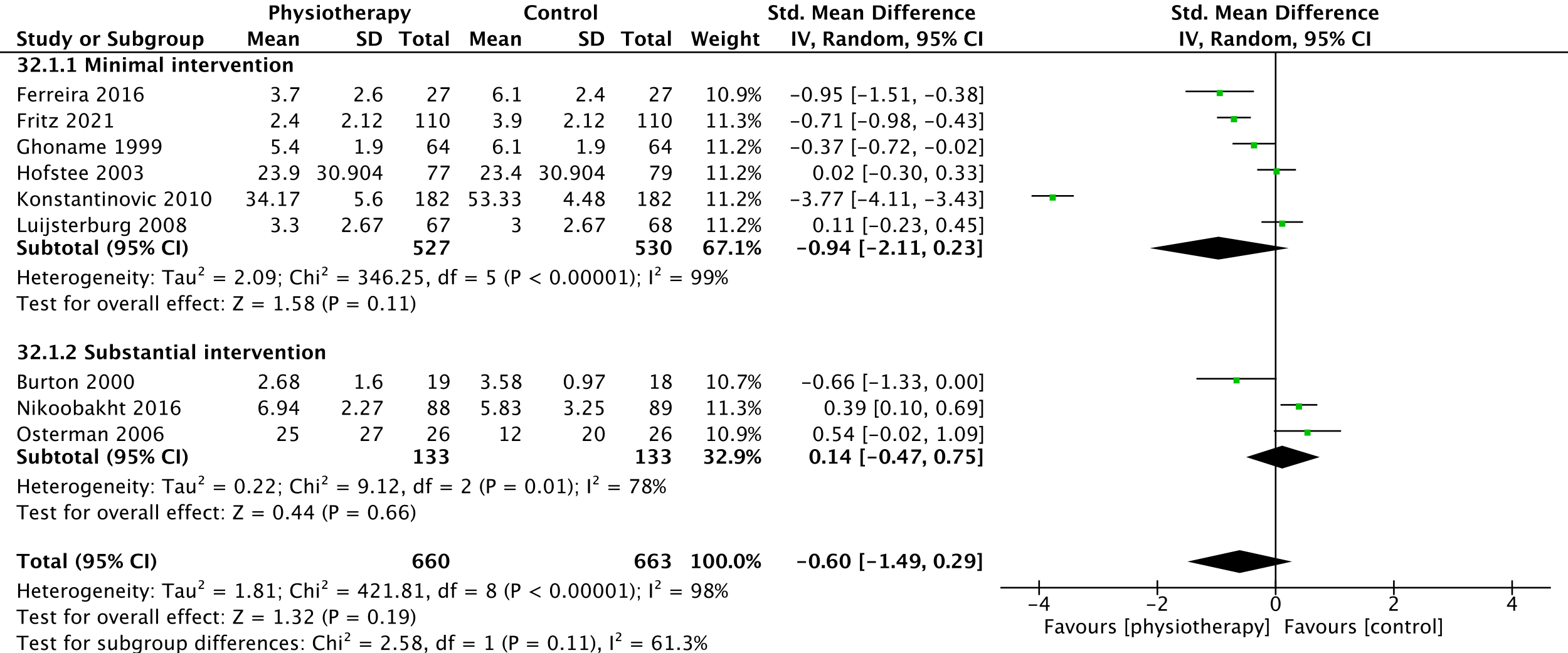


Supplemental Figure 1. Forest plot for primary outcome pain, short term, sensitivity analysis


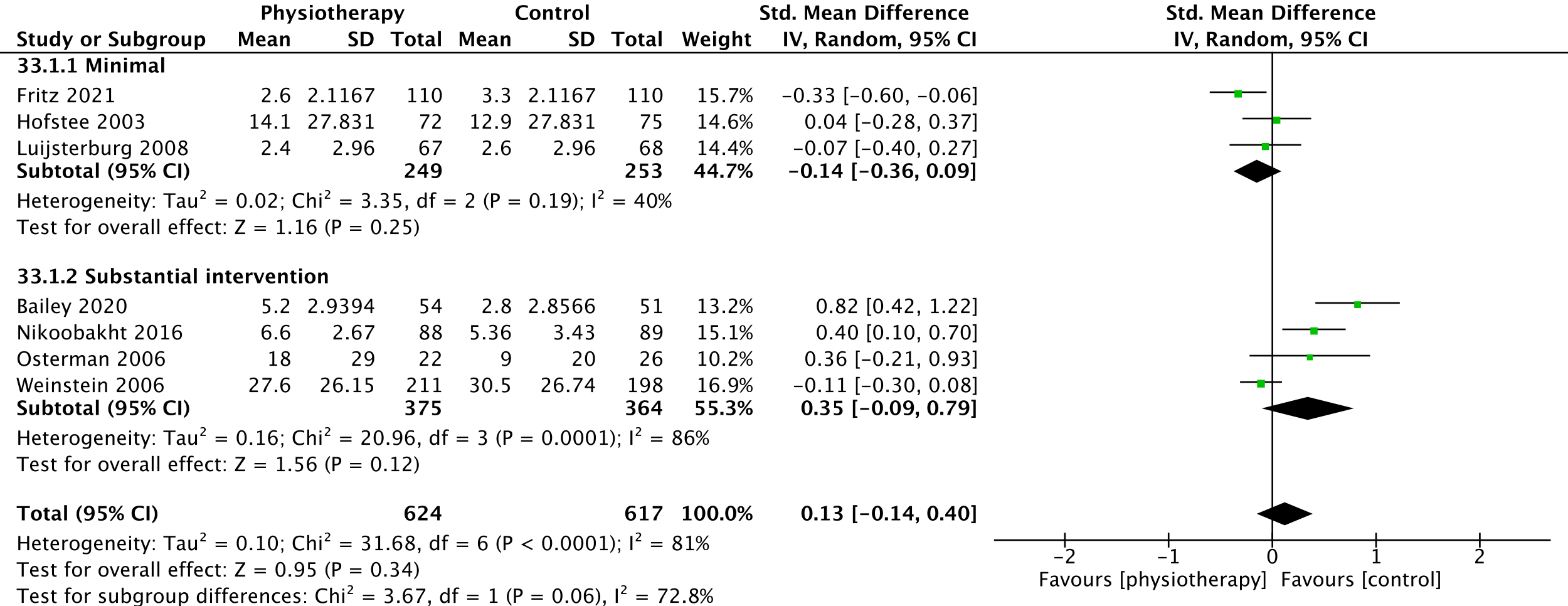


Supplemental Figure 2: Forest plot for primary outcome pain, medium term, sensitivity analysis


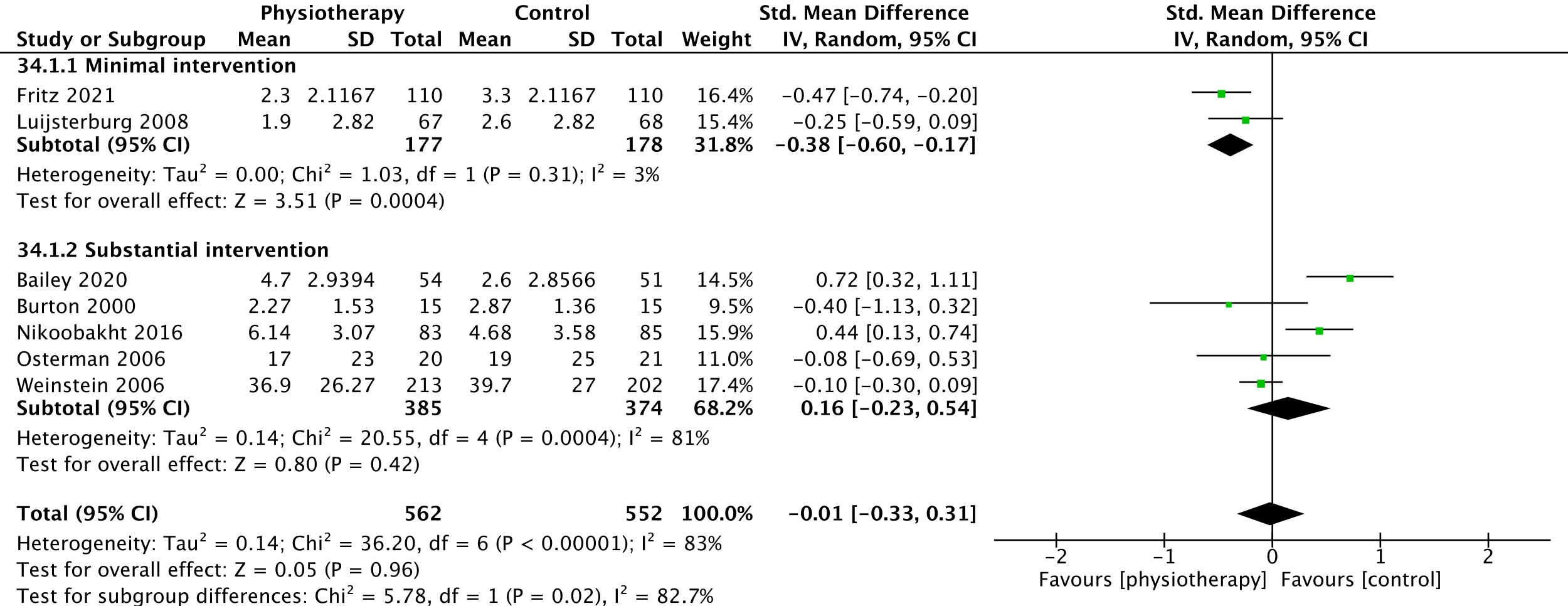


Supplemental Figure 3: Forest plot for primary outcome pain, long term, sensitivity analysis


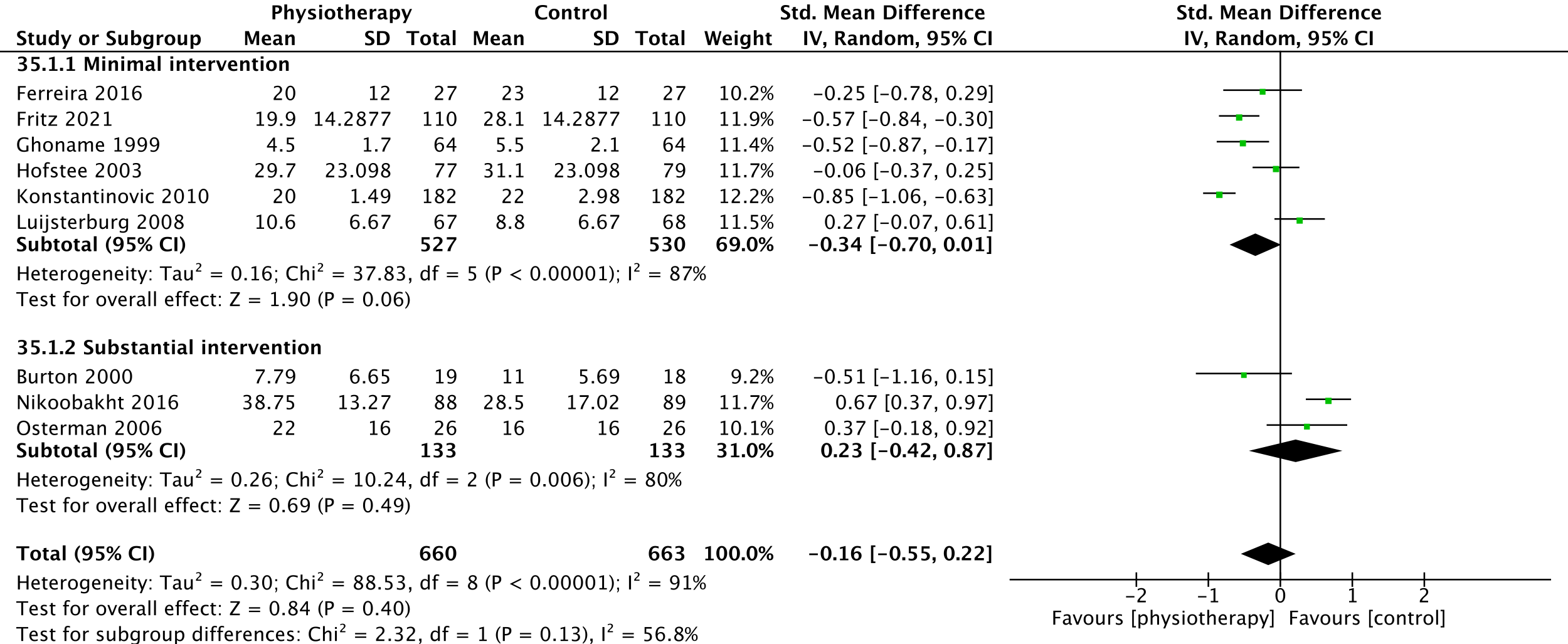
Supplemental Figure 4. Forest plot for primary outcome disability, short term, sensitivity analysis


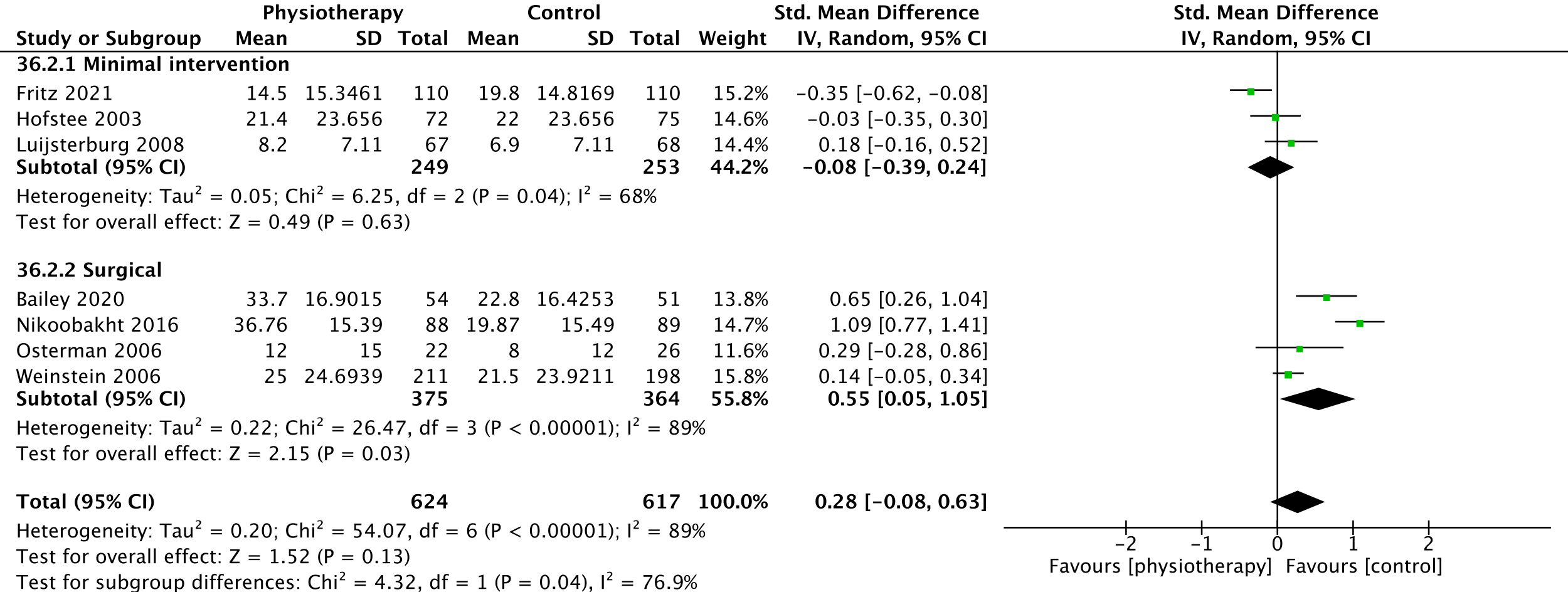


Supplemental Figure 5: Forest plot for primary outcome disability, medium term, sensitivity analysis


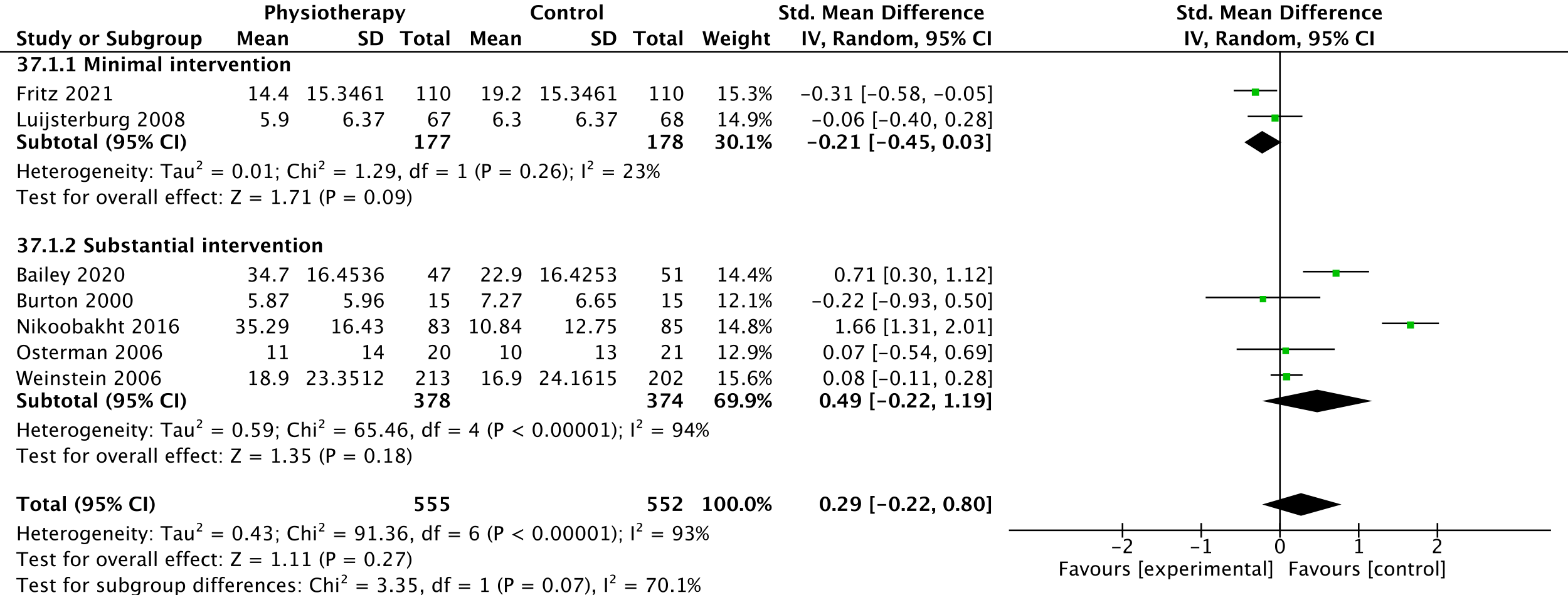


Supplemental Figure 6: Forest plot for primary outcome disability, long term, sensitivity analysis
