# Supplementary material for: How effective are physiotherapy interventions in treating people with sciatica? A systematic review and meta-analysis
Source: Eur Spine J. 2022 Dec 29;32(2):517–33. doi: 10.1007/s00586-022-07356-y (PMC9925551; doi:10.1007/s00586-022-07356-y)
Supplement: Supplementary file 2 — Supplementary file2 (DOCX 45 kb) [file 586_2022_7356_MOESM2_ESM.docx]

Supplemental Table 1. Example search strategies for all included databases.

Pubmed and CINAHL (EBSCO)

| S1 | sciatica [mh] OR sciatica OR (radicul* AND pain) OR radiculopathy [mh] OR radicul* OR (“nerve root*”AND disorder* OR compress* OR pain* OR inflam* OR irrita*) OR (nerve* OR neural) mechanosensit* |
| --- | --- |
| S2 | conservative* OR physical therapy modalities [mh] OR (“physical therap*” OR physiotherap* OR rehabilitation [mh] OR rehabil* OR manipulation, chiropractic [mh] OR manipulation osteopathic [mh] OR musculoskeletal manipulations [mh] OR “manual therap*” OR joint mobilization [mh] electrotherapy [mh] OR electrotherap* OR acupuncture [mh] OR dry needling [mh] or acupunctur* OR dry needl*” OR trigger point needl* OR exercise therapy [mh] or exercise [mh] OR exercise* OR “physical activ*” OR traction (spin* OR lumb*) OR massage [mh] OR massage OR “soft tissue therap*” OR self management [mh] |
| S3 | (mobilisation* OR mobilization*) AND (spin* OR joint* OR nerve* OR neural) |
| S4 | Randomized controlled trial [pt] OR controlled clinical trial [pt] OR randomized [tiab] OR placebo [tiab] OR clinical trials as topic [mesh: no exp] OR randomly [tiab] OR trial [ti] |
| S5 | Animals [mh] NOT humans [mh] |
| S6 | S4 NOT S5 |
| S7 | S2 OR S3 |
| S8 | S1 AND S6 AND S7 |

CENTRAL Trials (Cochrane)

| S1 | sciatic OR radiculopathy OR radicular pain OR nerve root OR neural mechanosensitivity OR nerve mechanosensitivity |
| --- | --- |
| S2 | Physical Therapy Modalities [MeSH] OR Self-Management [MeSH] OR Exercise Therapy [MeSH] OR Acupuncture Therapy [MeSH] |
| S3 | S1 AND S2 |

Embase

| S1 | Sciatica OR sciatica [mp] OR (raducul*[mp] AND pain[mp]) OR radiculopathy OR (nerve root* AND (disorder* OR compress* OR inflamm* OR irrita*))[mp] |
| --- | --- |
| S2 | (nerve OR neural)[mp] AND mechanosensit*[mp] |
| S3 | S1 OR S2 |
| S4 | Conservative treatment OR physiotherapy OR rehabilitation OR rehabil*[mp] OR (osteopathic manipulation OR chiropractic manipulation OR musculoskeletal manipulation OR spine manipulation) OR “manual therap*” OR musculoskeletal procedure |
| S5 | Mobilization |
| S6 | S2 AND S5 |
| S7 | (spine OR joint)[mp] |
| S8 | S5 AND S7 |
| S9 | Exp acupuncture OR exp electrotherapy OR kinesiotherapy OR traction therapy OR exp massage or exp self care |
| S10 | S4 OR S6 OR S8 OR S9 |
| S11 | S3 AND S10 |
| S12 | Limit S11 to (human and randomized controlled trials) |

Scopus

| S1 | sciatica OR “radicul* pain” OR radiculopathy OR (“nerve root* AND (disorder* OR compress* OR pain* OR inflamm* OR irrita*) OR ((nerve* OR neural) w/3 mechanosensit*)) |
| --- | --- |
| S2 | ((conservative* OR “physical therap*” OR physiotherap* OR rehabilita* OR “manual therap*” OR manipulation* W/3 (spinal OR joint)) OR mobilization W/3 (spinal OR joint OR nerve* OR neural)) OR (electrotherap* OR acupuncture OR “exercise therapy” OR massage OR “self management”) OR (traction W/3 (spine OR spinal OR lumbar))) |
| S3 | (LIMIT-TO (DOCTYPE, “ar”)) AND (LIMIT-TO (EXACTKEYWORD, “Human”) OR LIMIT-TO (EXACTKEYWORD, “Adult”) OR LIMIT-TO (EXACTKEYWORD, “Randomized Controlled Trial”) OR LIMIT-TO (EXACTKEYWORD, “Physical Therapy Modalities”) |

PEDro

| S1 | Sciatica OR lumbar radicul* OR lumbar radicular pain OR lumbar radiculopathy |
| --- | --- |
| S2 | Clinical trial |
| S3 | S1 AND S2 |

Supplemental Table 2. Diagnostic criteria

| **Study**  **Year**  **Country** | **Diagnostic criteria** |
| --- | --- |
| Physiotherapy vs minimal intervention (4 RCTs) | |
| Ferreira et al.^18^  2016  Brazil | Presence of neuropathic pain assessed by the Leeds Assessment of Neuropathic Symptoms and Signs (LANSS) score, score > or = 12. A neurological examination (manual muscle strength testing of the lower limbs, patellar and Achilles reflexes and sensation) participants with at least one positive neurological finding were classified as having nerve root compromise. |
| Fritz et al.^19^  2021  USA | Symptoms extending beyond the knee in past 72 hours, examination signs consistent with sciatica (positive result on straight leg raise test or sensory or motor deficit in a pattern consistent with a lumbar nerve root). |
| Ghoname et al.^20^  1999  USA | History of sciatica (defined as presence of constant or intermittent pain in one leg radiating below the knee, a positive straight-leg raising test, evidence of nerve-root compression at the L5-S1 level confirmed by radiologic testing). |
| Hofstee et al.^21^  2003  The Netherlands | Symptoms in one leg with at least one of: positive SLR < 60 degrees, myotomal weakness, dermatomal loss of sensation, reduced knee or ankle reflex, all limited to one or two adjoining nerve roots. |
| Konstantinovic et al.^22^  2010  Serbia | Clinical examination and additional neuroradiological and neurophysiological examinations. Criteria for radicular pain: typical dermatomal pain radiating beyond the knee toward the foot, pain evoked by stretching of the sciatic nerve and worsening on Valsalva manoeuvre, and signs of nerve root dysfunctions such as sensory, motor and reflex impairments. |
| Luijsterburg et al^23^  2008  The Netherlands | Presence of one of the following symptoms: more pain on coughing, sneezing or straining; decreased muscle strength in the leg; sensory deficit in the leg; positive straight leg raise test. |
| Santilli et al.^24^  2006  Italy | Moderate to severe radiating pain to one leg (5 or higher on VAS) and MRI evidence of disc protrusion with or without disc degeneration in the spinal segments involved in pain. Disc abnormalities classified according to the Modic classification and subjects with 4A herniated disc (protrusion with intact annulus) were included in the study. |
| Physiotherapy vs substantial intervention (10 RCTS) | |
| Amundsen et al.^25^  2000  Norway | Sciatic pain in the leg with or without pain in the back, together with radiologic signs of stenosis and compression of the clinically affected nerve root(s). |
| Bailey et al.^26^  2020  Canada | History of unilateral radiculopathy and findings on MRI of posterolateral herniation of the disc between the fourth and fifth lumbar vertebrae (L4-5) or in the lumbosacral junction (L5-S1) on the appropriate side, with compression of the corresponding nerve root. |
| Burton et al.^27^  2000  UK | Unilateral unremitting sciatica (leg pain worse than back pain). Positive straight leg raising test with positive nerve root tension signs, radiculopathy limited to single nerve root. Evidence of single-level non sequestrated lumbar disc herniation on either CT or MRI where imaging findings consistent with clinical picture. |
| Erginousakis et al.^28^  2011  Greece | Presence of pain of the appropriate quality with neurologic signs of radiculopathy. Small to medium sized intervertebral disk herniation on MRI that was symptomatic (leg pain with or without back pain; lancinating, burning, stabbing or electrical sensation of pain; straight leg raise limited to less than 30 degrees). |
| McMorland et al.^29^  2010  Canada | Leg dominant symptoms with objective signs of nerve root tethering +/- neurologic deficit with evidence of appropriate nerve root compression on MRI. |
| Mondal et al.^30^  2017  India | Lumbar disc herniation and unilateral radiculopathy with chronic LBP of more than 3 months with pain intensity limiting function and NRS score above 5. Only subjects with clinically, radiologically and neuro-physiologically diagnosed disc herniation at L3-4, L4-5 and L5-S1 were included. |
| Nikoobakht et al.^31^  2016  Iran | Diagnosis of lumbar radicular syndrome by attending neurologist (a chief complaint of leg pain, a positive SLR less than 60 degrees reproducing leg pain; and ipsilateral MRI confirmed disc herniation). |
| Osterman et al.^32^  2006  Finland | CT finding of intervertebral disc extrusion or sequester, and at least one specific physical finding (a positive straight leg raising test < 70 degrees, muscle weakness, altered deep tendon reflex, or a dermatomal sensory change). |
| Peul et al.^33^  2007  The Netherlands | Eligible patients had a radiologically confirmed disk herniation and had received a diagnosis from an attending neurologist of an incapacitating lumbosacral radicular syndrome. Correlation of MRI findings with symptoms was registered by the neurosurgeon. |
| Weber et al.^34^  1983  Norway | Clinical symptoms and signs of a fifth lumbar and/or first sacral root lesion, corresponded to the findings at radiculography. Radicular pain provoked by moderate exercise, by sitting position, or by increased abdominal pressure (coughing, sneezing or defecation). Other symptoms and signs were restricted mobility of the spine, defence scoliosis (tilt), positive SLR test, and/or persistent weakness of muscle groups. |
| Weinstein et al.^35^  2006  USA | Radicular pain and evidence of nerve-root tension sign (SLR positive between 30 and 70 degrees or positive femoral tension sign) or a corresponding neurologic deficit (asymmetrical depressed reflex, decreased sensation in a dermatomal distribution, or weakness in a myotomal distribution). Imaging (MRI or CT) showing disk herniation at a level and side corresponding to the clinical symptoms. |

Supplemental Table 3. Results of secondary outcome measures

| Study  Year  Country | Secondary outcome measures and timepoints of interest | Results Physiotherapy intervention G1* | Results Control intervention G2* |
| --- | --- | --- | --- |
| Physiotherapy vs minimal intervention (7 RCTs) | | | |
| Ferreira et al.^18^  2016  Brazil | GPE(-5 to 5) 4w  Treatment adherence 2w | GPE short 2(1.5)  85% compliance | GPE short -0.7(1.9)  n/a |
| Fritz et al.^19^  2021  USA | EQ-5D 4w, 6m, 12m  FABQ 4w, 6m, 12m | QoL short 0.76 (95%CI 0.73, 0.8)  QoL med 0.8 (95%CI 0.77, 0.84)  QoL long 0.82 (95%CI 0.79, 0.86)  Psych short 9.5(95%CI 8.3, 10.7)  Psych med 8.9 (95%CI 7.6,10.1)  Psych long 7.1 (95%CI 5.9, 8.4) | QoL short 0.7 (95%CI 0.66, 0.73)  QoL med 0.79 (95%CI 0.75,0.82)  QoL long 0.78 (95% CI 0.74,0.81)  Psych short 11.3 (95%CI 10.1,12.5)  Psych med 9.6 (95%CI 8.3,10.8)  Psych long 10.7 (95%CI 9.5,12.0) |
| Ghoname et al.^20^  1999  USA | None reported |  |  |
| Hofstee et al.^21^  2003  The Netherlands | None reported |  |  |
| Konstantinovic  et al.^22^  2010  Serbia | SF-12 mental component 3w | Psych short median 18  (IQR 17-19) | Psych short median 17  (IQR15-18) |
| Luijsterburg et al^.23^  2008  The Netherlands | GPE dichotomised  6w, 12w, 12m  TSK 12m | GPE short 38/67 (60%)  GPE med 47/67 (70%)  GPE long 53/67 (79%)  Psych long 35.7 (7.11) | GPE short 30/68 (44%)  GPE med 42/68 (62%)  GPE long 38/68 (56%)  Psych long 36.5 (7.11) |
| Santilli et al.^24^  2006  Italy | None reported |  |  |
| Physiotherapy vs substantial intervention (11 RCTS) | | | |
| Amundsen et al.^25^  2000  Norway | None reported |  |  |
| Bailey et al.^26^  2020  Canada | SF-36 mental component 6m, 12m | Psych med 42.2 (1.6SE)  Psych long 42.3 (1.6SE) | Psych med 48.6 (1.6SE)  Psych long 48.1 (1.6SE) |
| Burton et al.^27^  2000  UK | None reported |  |  |
| Erginousakis et al.^28^  2011  Greece | None reported |  |  |
| McMorland et al.^29^  2010  Canada | SF-36 mental health 6w | Psych short 78.6 (11.6) | Psych 77.7 (16.8) |
| Mondal et al.^30^  2017  India | None reported |  |  |
| Nikoobakht et al.^31^  2016  Iran | SF-36 mental component 1m, 3m, 12m | Psych short 37.16 (26.06)  Psych med 38.98 (26.81)  Psych long 39.43 (27.49) | Psych short 41.68 (21.46)  Psych med 44.99 (24.75)  Psych long 45.8 (25.43) |
| Osterman et al.^32^  2006  Finland | DEPS 6w,12m  SLR positive 6w, 12m | Psych short 6 (5)  Psych long 3 (4)  Neurological function short 11/26  Neurological function long 3/20 | Psych short 6 (5)  Psych long 3 (4)  Neurological function short 5/26  Neurological function long 1/21 |
| Peul et al.^33^  2007  The Netherlands | GPR 8w, 6m, 12m  SF-36 mental component 8w, 6m, 12m | GPR short 3.1(0.1SE)  GPR med 2.3 (0.1SE)  GPR long 2.1 (0.1SE)  Psych short 73.0 (1.7SE)  Psych med 80.5 (1.5SE)  Psych long 81.1 (1.4SE) | GPR short 2.2 (0.1SE)  GPR med 2.1 (0.1SE)  GPR long 1.9 (0.1SE)  Psych short 82.1 (1.3SE)  Psych med 83.2 (1.3SE)  Pysch long 83 (1.3SE) |
| Weber et al.^34^  1983  Norway | None reported |  |  |
| Weinstein et al.^35^  2006  USA | None reported |  |  |

RCT Randomised Controlled Trial; G Group; SD Standard Deviation; FABQ Fear Avoidance Beliefs Questionnaire; Psych = Pyschological; QDS Quebec Disability Scale; TSK Tampa Scale of Kinesiophobia; GPE Global Perceived Effect; SF-36 36 Item Short Form Survey; McGill PRI(R) McGill Pain Rating Index Rank value; 15D QoL Health related Quality of Life; DEPS Depression Scale; SLR Straight Leg Raise; GPR Global Perceived Rating scale (lower score represents recovery), SE: standard error; CI: confidence interfal; med = medium; m = months; IQR Inter Quartile Range

*data are reported as mean (SD) unless stated otherwise stated

Supplemental Table 4. Adverse Events

| **Study**  **Year**  **Country** | **Adverse events physiotherapy intervention** | **Adverse events control intervention** |
| --- | --- | --- |
| Physiotherapy vs minimal intervention (7 RCTs) | | |
| Ferreira et al.^18^  2016  Brazil | One participant did vigorous hamstring stretching instead of sliding and tensioning exercise and reported an adverse effect, symptoms subsided within 24 hours. |  |
| Fritz et al.^19^  2021  USA | 37 non-serious side effects of back pain/stiffness |  |
| Ghoname et al.^20^  1999  USA | Unreported* | Unreported* |
| Hofstee et al.^21^  2003  The Netherlands | 1 cauda equina syndrome |  |
| Konstantinovic  et al.^22^  2010  Serbia | Persistent worsening of pain (10 consecutive days) in 1 patient  Transitional worsening of pain (maximum duration 6 hours) in 27 of 182 patients. | Persistent worsening of pain (10 consecutive days) in 1 patient |
| Luijsterburg et al.^23^  2008  The Netherlands | Unreported* | Unreported* |
| Santilli et al.^24^  2006  Italy | Unreported* | Unreported* |
| Physiotherapy vs surgical (11 RCTS) | | |
| Amundsen et al.^25^  2000  Norway | Unreported* | Unreported* |
| Bailey et al.^26^  2020  Canada |  | Dural tear, wound infection, nerve-root injury, post-operative adjacent level condition, new onset post-operative neuropathic pain, recurrent herniation after surgery. |
| Burton et al.^27^  2000  UK |  | No major complications |
| Erginousakis et al.^28^  2011  Greece | Unreported | Unreported |
| McMorland et al.^29^  2010  Canada |  | No major complications |
| Mondal et al.^30^  2017  India | Unreported | Unreported |
| Nikoobakht et al.^31^  2016  Iran |  | No major complications |
| Osterman et al.^32^  2006  Finland |  | Urosepsis |
| Peul et al.^33^  2007  The Netherlands |  | 2 dural tears, 1 wound haematoma |
| Weber et al.^34^  1983  Norway | Unreported* | Unreported* |
| Weinstein et al.^35^  2006  USA |  | 10 cases of dural tear/spinal fluid leak, 1 vascular injury, 2 other undefined intraoperative complications, 4 postsurgical superficial wound infections, 9 other undefined postsurgical complications/events, 9 additional surgeries reoperated at one year, 5 recurrent herniations and 4 other undefined complications. |

*pre-dates publication of Consort Guidelines 2010
